# Supplementary material for: Complex interplay between gene deletions and the environment uncovers cellular roles for genes of unknown function in Escherichia coli
Source: mSystems. 2025 Jun 10;10(7):e00206-25. doi: 10.1128/msystems.00206-25 (PMC12282094; doi:10.1128/msystems.00206-25)
Supplement: Supplemental Material — Supplemental figures and tables. [file msystems.00206-25-s0002.pdf]

Supplemental material

Complex interplay between gene deletions and the environment uncovers cellular roles for genes of unknown function in *Escherichia coli*

Running title: Nutrient-dependent phenotypic variability of *E. coli* deletion strains

Kaat Sondervorst, Kristina Nesporova, Matthew Herdman, Bart Steemans, Joëlle Rosseels, Sander K. Govers

Supplemental figures

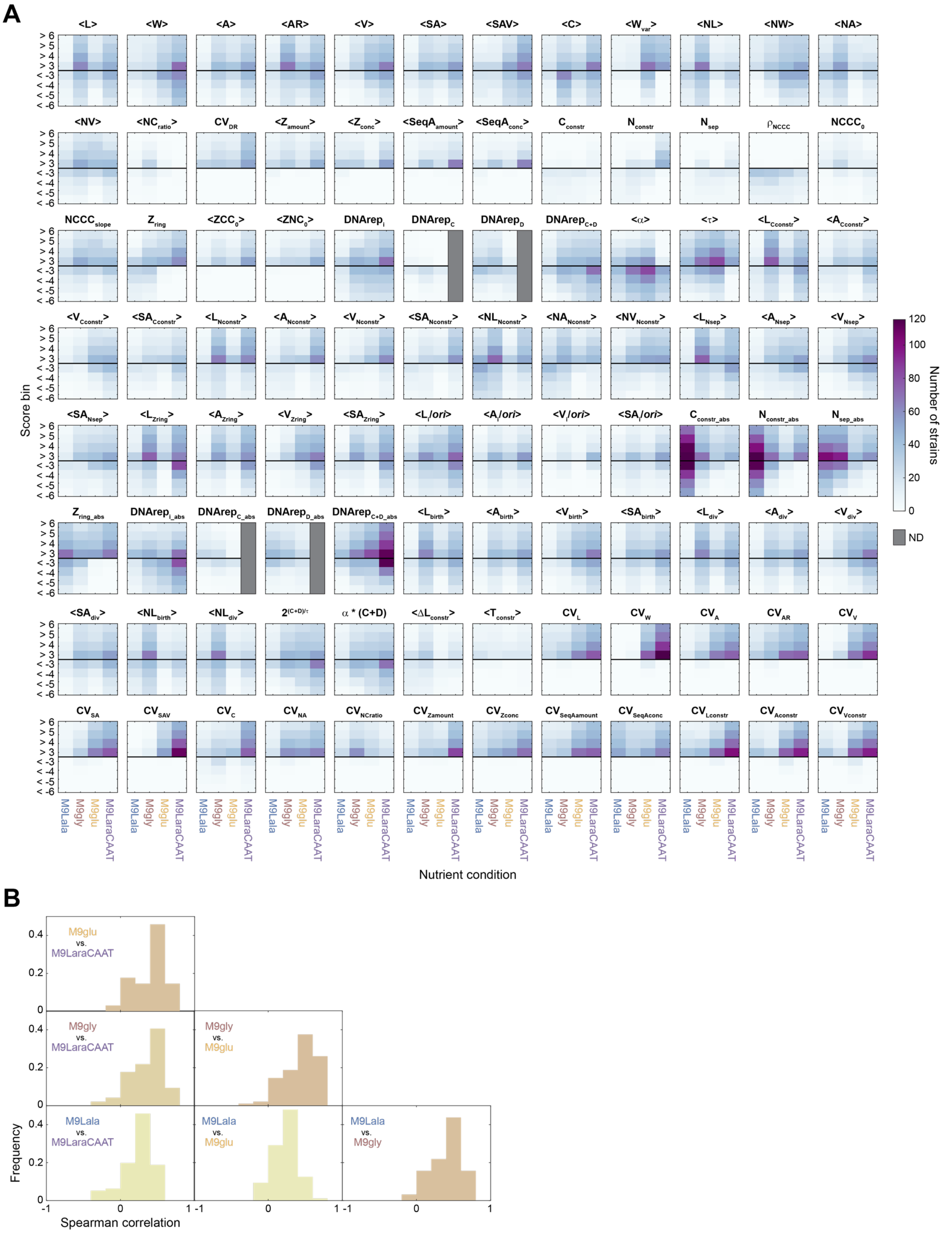

Figure S1. Cross-condition image-based profiling dataset.

A. Heatmaps showing the distribution of deviating normalized scores ($|s| \geq 3$) for each phenotypic feature. Feature abbreviations are defined in the legend of Dataset S1. Grey boxes indicate DNA replication-related features that could not be determined (ND) because of the presence of more than two overlapping rounds of DNA replication.

B. Frequency distributions of the pairwise correlations of deviating phenotypes ($|s| \geq 3$) across nutrient conditions. For each feature, only deletion strains displaying a deviating phenotype ($|s| \geq 3$) in at least one nutrient condition were considered upon calculation of the correlation coefficients.

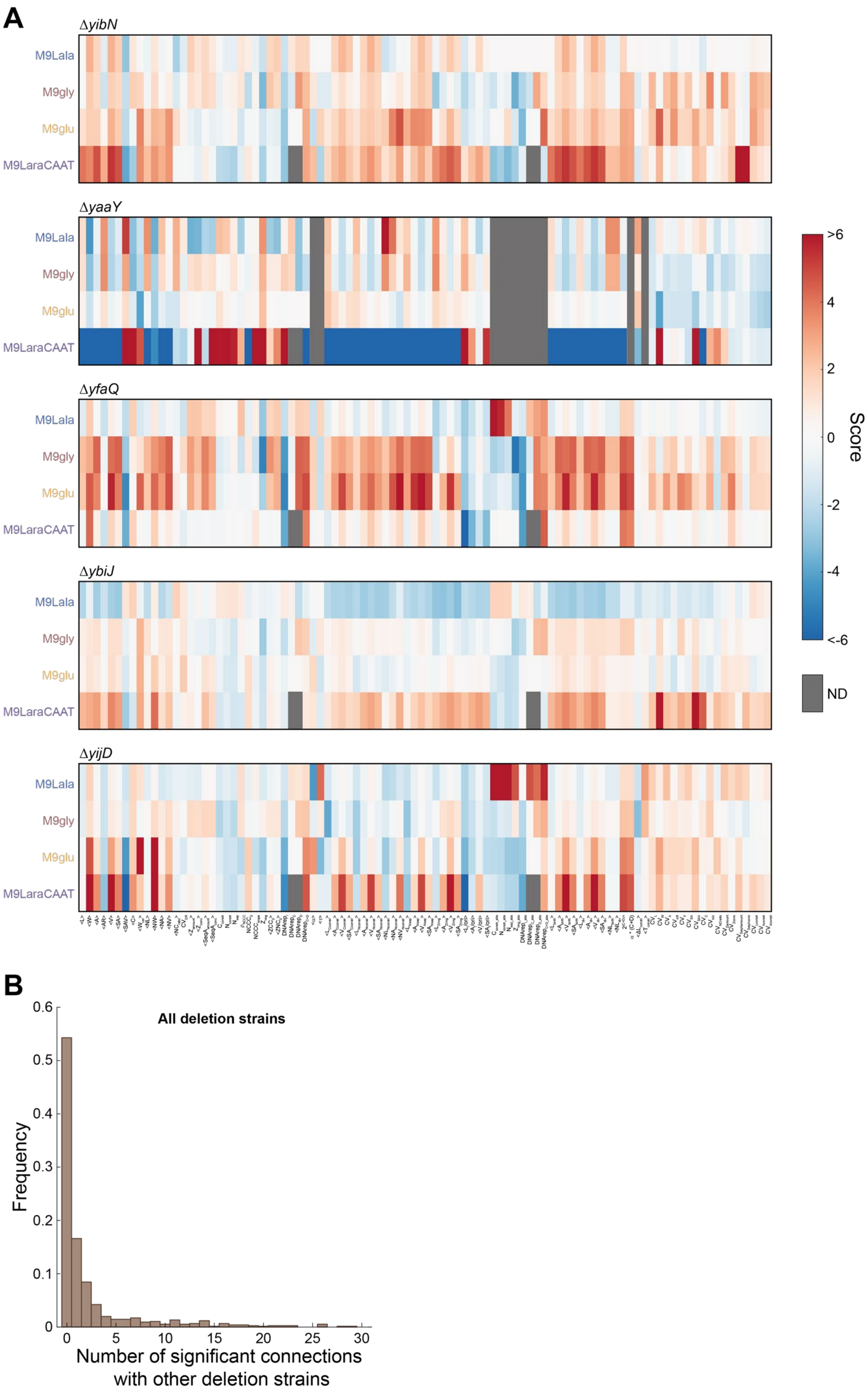
A
ΔyibN
ΔyaaY
ΔyfaQ
ΔybiJ
ΔyijD
M9Lala
M9gly
M9glu
M9LaraCAAT
>6
4
2
0
-2
-4
<-6
Score
ND
B
All deletion strains
Frequency
0.6
0.5
0.4
0.3
0.2
0.1
0
0
5
10
15
20
25
30
Number of significant connections with other deletion strains

Figure S2. Y-gene deletion phenoprints.

A. Heatmap showing the phenoprints of the Δ*yibN*, Δ*yaaY*, Δ*yfaQ*, Δ*ybiJ*, and Δ*yijD* strains. The phenoprints consist of the normalized scores for each of the 96 extracted population level features across four nutrient conditions. Grey boxes indicate DNA replication-related features that could not be determined (ND).

B. Frequency distribution of the number of significant phenoprint connections between all deletion strains and other deletion strains.

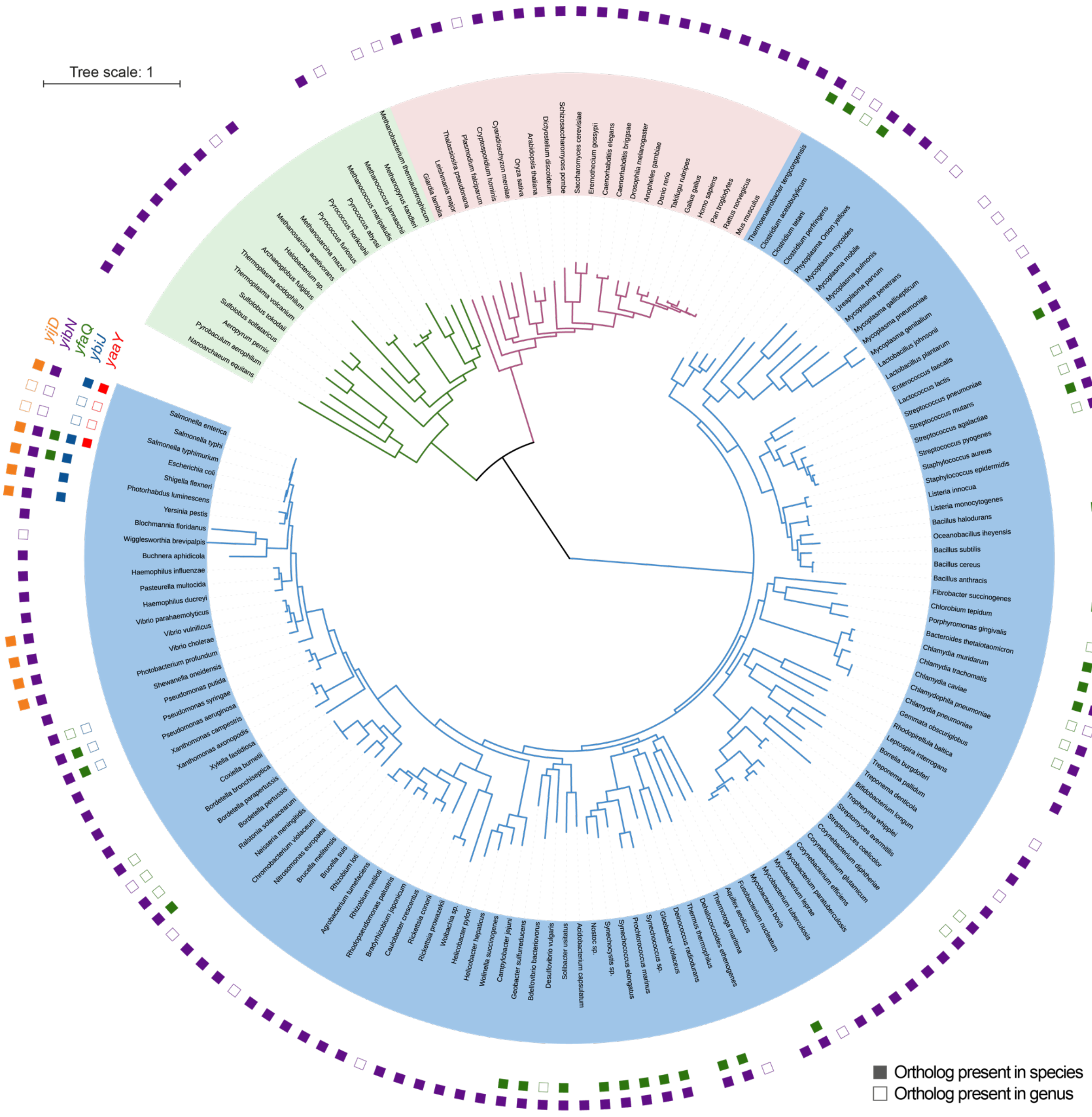

Figure S3. Conservation of selected y-genes across the tree of life.

The presence of the indicated y-gene orthologs, determined using the EggNOG database, was mapped onto the tree of life. Full squares indicate the presence of the respective orthologue in a specific species, while empty square indicate the presence of the orthologue in the relevant genus of the species present in the tree (but not in that species itself).

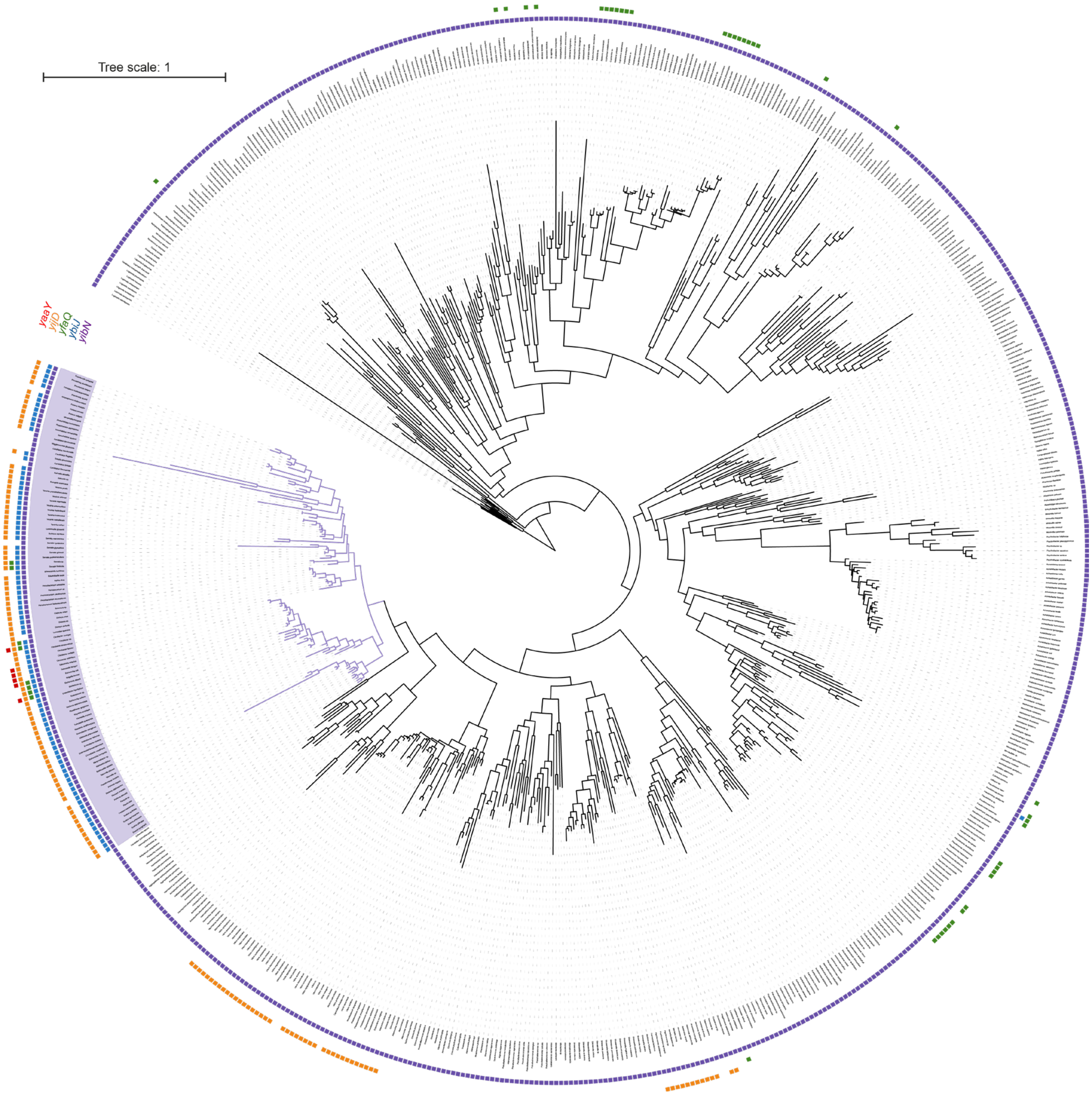

Figure S4. Conservation of selected y-genes across the Proteobacteria.

Full squares indicate the presence of the respective orthologue in a specific species.

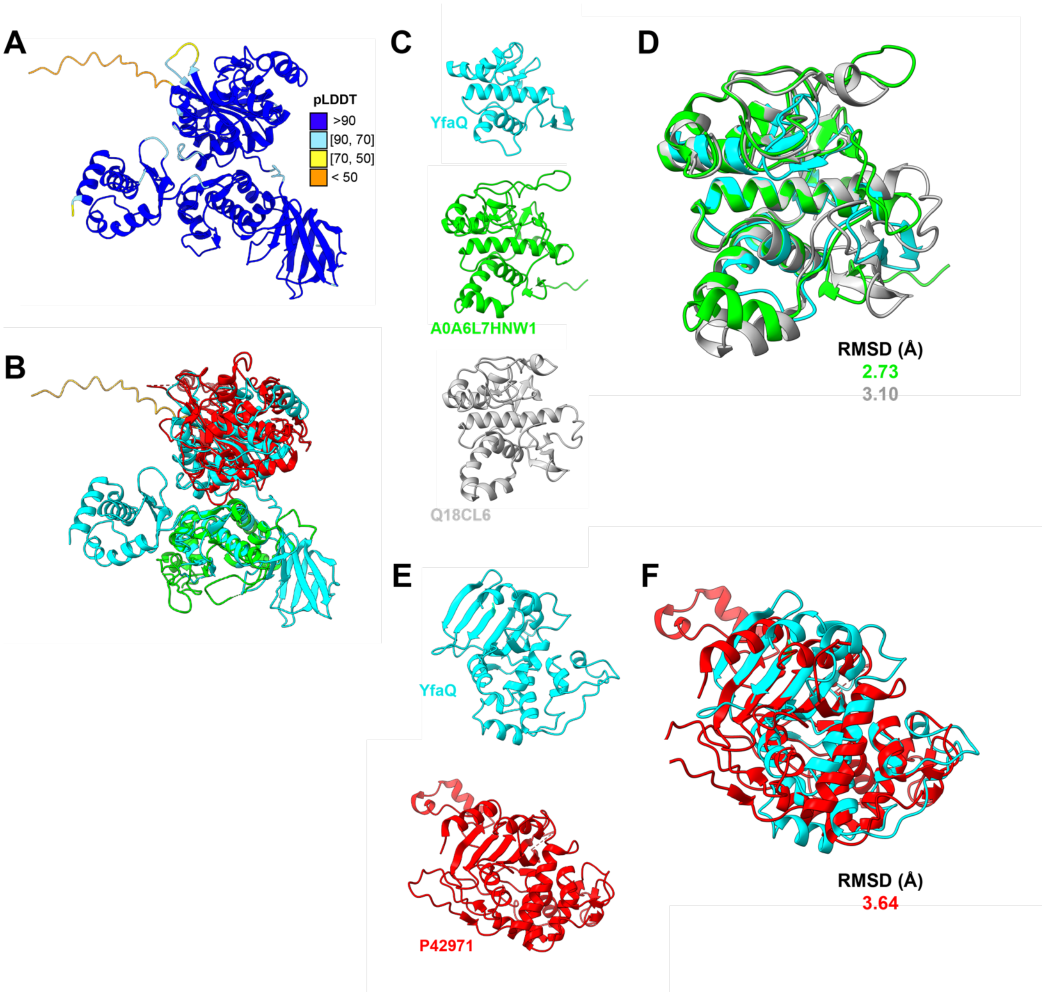

Figure S5. YfaQ structural predictions suggest a bipartite arrangement.

A. Structural depiction (ribbon) of YfaQ generated by AlphaFold 2 and coloured by pLDDT. The low pLDDT C-terminal peptide comprises the predicted secretion signal.

B. Superimposed structures of YfaQ (AF2) (cyan) with the homologous domains from the PBP3 protein P42971 (PDB 7BN9) from *Bacillus subtilis* (red) and the SpoIID protein A0A6L7HNW1 (PDB 4RWR) from *Bacillus anthracis* (green).

C. Structural depictions of the PBP domains of YfaQ (AF2; residues 320-445) (top; cyan), A0A6L7HNW1 (PDB 4RWR) (middle; green), and Q18CL6 (PDB 7RD0, from *Clostridioides difficile*) (bottom; gray).

D. Superimposed structural depictions between PBP domains showing structural similarity. The three central α-helices comprising the (putative) enzymatic cores of A0A6L7HNW1 and Q18B92 show particularly strong structural similarity with the predicted structure of YfaQ. The low RMSDs indicate the good quality of the alignments.

E. Structural depictions of the SpoIID domains of YfaQ (AF2; residues 24-245) (cyan) and P42971 (PDB 7BN9) (red).

F. Superimposed structural depictions of SpoIID domains showing structural similarity. The low RMSD indicates the good quality of the alignments.

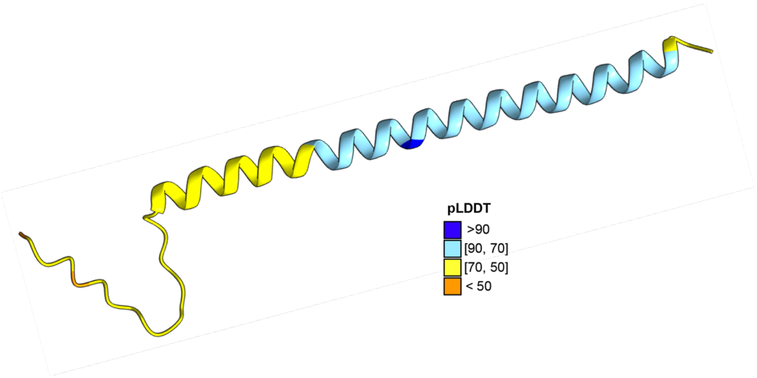

Figure S6. Predicted structure of YaaY.

Structural depiction (ribbon) of YaaY generated by AlphaFold 2 and coloured by pLDDT.

Supplemental tables

Table S1. ***E. coli*** **sequence types with limited conservation of** ***yfaQ*****.**

Sequence type	Total number of genomes	Number of genomes without *yfaQ*	Fraction without yfaQ
ST33	857	722	0.842473746
ST349	387	387	1
ST10	7513	190	0.025289498
ST542	231	177	0.766233766
ST278	328	106	0.323170732
ST90	368	97	0.263586957
ST3595	68	68	1
ST131	10228	54	0.005279625
ST1176	43	43	1
ST48	928	34	0.036637931
ST86	127	28	0.220472441
ST155	1490	25	0.016778523
ST59	318	25	0.078616352
ST12	834	21	0.025179856
ST2914	20	20	1
ST206	460	19	0.041304348
ST120	35	18	0.514285714
ST226	137	17	0.124087591
ST871	47	17	0.361702128
ST345	282	15	0.053191489
ST216	238	12	0.050420168
ST6786	12	12	1
ST2617	11	11	1
ST9700	11	11	1
ST5523	12	10	0.833333333
ST718	272	10	0.036764706

Table S2. Structural homology determinations of YfaQ, YibN, YbiJ, and YijD.

Protein	UniProt ID	Hit	UniProt ID	PDB ID	Name	Organism	Probability	E-value	RMSD (Å)	TM-score
**YfaQ**	P76463	1	P42971	7BN9	Penicillin-binding protein (PBP3)	*Bacillus subtilis*	99.86	7.6E-21	3.64	0.71
		2	A0A6L7HNW1	4RWR	Stage II sporulation protein D	*Bacillus anthracis*	99.86	8.8E-21	2.73	0.46
		3	Q18B92	7RD0	Penicillin-binding protein (PBP3)	*Clostridioides diffiicle*	99.86	1.3E-20	3.80	0.71
		4	M1RM73	8U55	Penicillin-binding protein (PBP5)	*Enterococcus faecium*	99.86	1.5E-20	3.60	0.72
		5	Q93IC2	1VQQ	Penicillin-binding protein (PBP2)	*Staphylococcus aureus*	99.85	3.4E-20	3.58	0.72
		6	A0A0H2WVW5	5TRO	Penicillin-binding protein (PBP1)	*Staphylococcus aureus*	99.85	4.4E-20	3.73	0.72
		7	G1C6X4	7ZG8	Penicillin-binding protein (PBP2)	*Acinetobacter baumannii*	99.84	2.0E-20	3.77	0.71
		8	Q18CL6	5TXU	Stage II sporulation protein D	*Peptoclostridium difficile*	99.84	4.7E-21	3.10	0.48
		9	P0AD68	4BJP	Penicillin-binding protein (PBP3)	*Escherichia coli*	99.84	5.5E-20	3.81	0.71
		10	C8W8H7	4R23	Peptidoglycan glycosyltransferase	*Atopobium parvulum*	99.84	4.1E-20	3.83	0.70
**YbiJ**	P0AAX3	1	Q57SJ8	2NOC	Putative periplasmic protein	*Salmonella enterica*	99.94	7.7E-26	2.17	0.74
		2	Q7CR88	2JNA	Putative secreted protein	*Salmonella enterica*	99.94	3.0E-25	1.67	0.77
		3	Q8ZPL1	2M2J	Putative periplasmic protein	*Salmonella enterica*	99.89	1.3E-22	1.53	0.83
		4	Q7CR49	2MA4	Putative periplasmic protein	*Salmonella enterica*	99.84	5.3E-20	2.20	0.75
		5	Q8ZPL1	4EVU	Putative periplasmic protein (YdgH)	*Salmonella enterica*	99.83	1.6E-19	1.52	0.80
		6	Q7CR88	2MA8	Putative secreted protein	*Salmonella enterica*	99.80	1.6E-18	1.54	0.81
		7	P69411	6T1W	Outer membrane lipoprotein (RcsF)	*Escherichia coli*	99.80	7.3E-03	3.37	0.41
		8	P69411	2Y1B	Outer membrane lipoprotein (RcsF)	*Escherichia coli*	94.90	6.1E-01	1.84	0.81
		9	P69411	9BIY	Outer membrane lipoprotein (RcsF)	*Escherichia coli*	94.22	5.2E-01	3.46	0.37
		10	P69411	2L8Y	Outer membrane lipoprotein (RcsF)	*Escherichia coli*	93.17	1.4E+00	1.41	0.84
**YibN**	P0AG27	1	C8WS08	3TP9	Beta-lactamase and Rhodanese domain protein	*Alicyclobacillus acidocaldarius*	99.41	9.4E-12	1.54	0.84
		2	A0A2R2W8Z3	8K55	Sulfurtransferase	*Frondihabitans sp. 762G35*	99.41	3.3E-11	1.98	0.84
		3	Q9I0N4	1YT8	Thiosulfate sulfurtransferase	*Pseudomonas aeruginosa*	99.40	1.8E-11	2.04	0.80
		4	E2QRA0	3P3A	Thiosulfate sulfurtransferase	*Mycobacterium thermoresistible*	99.38	4.0E-11	2.08	0.82

		5	P9WHF9	3AAY	Thiosulfate sulfurtransferase	*Mycobacterium tuberculosis*	99.30	1.4E-10	1.91	0.84
		6	Q5ZRP2	4F67	Sulfurtransferase	*Legionella pneumophila*	99.27	1.0E-10	2.35	0.73
		7	Q56748	1QXN	Sulfide dehydrogenase	*Wolinella succinogenes*	99.27	2.6E-09	2.40	0.72
		8	P52197	1E0C	Sulfurtransferase	*Azotobacter vinelandii*	99.24	1.4E-09	1.81	0.82
		9	Q5NFU2	6MXV	Rhodanese-like family protein	*Francisella tularensis*	99.21	1.9E-09	2.91	0.65
		10	Q5SJI0	1UAR	Sulfurtransferase	*Thermus thermophilus*	99.20	1.7E-09	3.05	0.71
**YijD**	P0AF40	1	P68806	3HZQ	Large-conductance mechanosensitive channel	*Staphylococcus aureus*	78.30	9.8E+00	4.63	0.40
		2	Q5L1G5	5OQT	Amino acid transporter	*Geobacillus kaustophilus*	73.14	5.1E+01	4.85	0.42
		3	P60061	7O82	Arginine/agmatine antiporter	*Escherichia coli*	65.15	8.6E+01	4.17	0.49
		4	A8UCQ5	6F2G	Putative amino acid/polyamine transport protein	*Cornobacterium sp. AT7*	64.56	8.7E+01	4.64	0.46
		5	P0ADZ7	2RDD	Acriflavine resistance protein B	*Escherichia coli*	62.67	1.5E+01	5.20	0.39
		6	Q8TNK0	4Y7K	Large-conductance mechanosensitive channel	*Methanosarcina acetivorans*	55.21	2.9E+01	4.79	0.36
		7	N/A	7QOA	Cytosine permease; Membrane Transporter	*Proteus vulgaris*	54.29	1.5E+02	4.62	0.46
		8	E4KPW4	5M87	Divalent metal cation transporter MntH	*Eremococcus coleocola*	51.59	1.1E+02	5.00	0.47
		9	Q9KP76	6MJP	ABC-transporter; Lipopolysaccharide transport	*Vibrio cholerae*	49.71	2.7E+01	4.58	0.34
		10	A5U127	2OAR	Large-conductance mechanosensitive channel	*Mycobacterium tuberculosis*	49.41	4.2E+01	4.60	0.32

Protein IDs and amino acid sequences were taken from UniProt. For homology prediction, sequences were subjected to HHpred through the MPI Bioinformatics Toolkit (https://toolkit.tuebingen.mpg.de/) (2); searches were carried out against the PDB_mmCIF70 database. For each search, the top 10 hits were taken for further validation and are listed along with their UniProt and PDB IDs. The related protein and organism names are based on UniProt entries. Probability and E-values were taken directly from HHPred to assess the quality of the homology prediction (higher values indicate higher confidence). Root mean square deviation (RMSD) and template modeling (TM) scores were calculated using the pairwise structural alignment tool from RSCB-PDB (https://www.rcsb.org/alignment) using the TM-align alignment method (3). Lower RMSD and higher TM-scores indicate better alignments between structures. For YfaQ, protein alignments were restricted to the relevant domain, i.e. residues 1-245 for PBP and peptidoglycan glycotransferase proteins and residues 246-549 for SpoIID proteins, as each HHpred hit aligned to only one of the two domains.

Table S3. Construction of strains used in this study. All modifications were verified by both PCR and sequencing.

Strains	Construction method
E. coli MG1655 *yfaQ*::*yfaQ-msfgfp*	This strain was constructed using *E. coli* K12 MG1655 as a parental strain. An *msfgfp-frt-kan^R-frt* amplicon flanked by short (50 bp) nucleotide sequences homologous to its target region (50 nt on both sides of the *yfaQ* stop codon) was generated by PCR amplification using oligonucleotides P1 and P2, and pDHL1029-msfGFP (4) as a template. This amplicon was used to replace the stop codon of the native *yfaQ* gene, yielding the chromosomal *yfaQ-msfgfp* fusion. Successful recombination was verified by colony PCR using oligonucleotides P9 and P10. The kanamycin resistance cassette was subsequently excised by transiently equipping this strain with plasmid pCP20 expressing the Flp site-specific recombinase (5), after which oligonucleotides P9 and P10 were used to generate a colony PCR product that was sent for sequencing.
E. coli MG1655 *yibN*::*yibN-msfgfp*	This strain was constructed using *E. coli* K12 MG1655 as a parental strain. An *msfgfp-frt-kan^R-frt* amplicon flanked by short (50 bp) nucleotide sequences homologous to its target region (50 nt on both sides of the *yibN* stop codon) was generated by PCR amplification using oligonucleotides P3 and P4, and pDHL1029-msfGFP (4) as a template. This amplicon was used to replace the stop codon of the native *yibN* gene, yielding the chromosomal *yibN-msfgfp* fusion. Successful recombination was verified by colony PCR using oligonucleotides P11 and P12. The kanamycin resistance cassette was subsequently excised by transiently equipping this strain with plasmid pCP20 expressing the Flp site-specific recombinase (5), after which oligonucleotides P11 and P12 were used to generate a colony PCR product that was sent for sequencing.
E. coli MG1655 *ybiJ*::*ybiJ-msfgfp*	This strain was constructed using *E. coli* K12 MG1655 as a parental strain. An *msfgfp-frt-kan^R-frt* amplicon flanked by short (50 bp) nucleotide sequences homologous to its target region (50 nt on both sides of the *ybiJ* stop codon) was generated by PCR amplification using oligonucleotides P5 and P6, and pDHL1029-msfGFP (4) as a template. This amplicon was used to replace the

	stop codon of the native *ybiJ* gene, yielding the chromosomal *ybiJ-msfgfp* fusion. Successful recombination was verified by colony PCR using oligonucleotides P13 and P14. The kanamycin resistance cassette was subsequently excised by transiently equipping this strain with plasmid pCP20 expressing the Flp site-specific recombinase (5), after which oligonucleotides P13 and P14 were used to generate a colony PCR product that was sent for sequencing.
E. coli MG1655 *yijD*::*yijD-msfgfp*	This strain was constructed using *E. coli* K12 MG1655 as a parental strain. An *msfgfp-frt-kan^{R}-frt* amplicon flanked by short (50 bp) nucleotide sequences homologous to its target region (50 nt on both sides of the *yijD* stop codon) was generated by PCR amplification using oligonucleotides P7 and P8, and pDHL1029-msfGFP (4) as a template. This amplicon was used to replace the stop codon of the native *yijD* gene, yielding the chromosomal *yijD-msfgfp* fusion. Successful recombination was verified by colony PCR using oligonucleotides P15 and P16. The kanamycin resistance cassette was subsequently excised by transiently equipping this strain with plasmid pCP20 expressing the Flp site-specific recombinase (5), after which oligonucleotides P15 and P16 were used to generate a colony PCR product that was sent for sequencing.

Table S4. Oligonucleotides used in this study.

Name	Sequence (5' to 3')
P1	cgaaagcgccaccagtaacaacggaagaaaaatctttcgcatagcaattccggggatccgtcgacc
P2	ggcttgatgagaactatatcgaaacgctgacccgacaattattaatggacagcggtggcggtggc
P3	gggggcctaattgcagctaacggcctgcatcatgaaagacgacaggtaaaattccggggatccgtcgac
P4	ggcgtcgctggctgggctggcgaaaacttgcctttggtgcgcggcaaaagcggtggcggtggcagtaaag
P5	atctgttgccggatgcggcgtaaacgccttatccgtcctacgaaattccggggatccgtcgacc
P6	ccaccaacaacaataaattaagcggtactgcggtaatttataagagcggtggcggtggc
P7	tactgaccgcgaagtaaaacccatgccggatgcgccagcatccggcataataccgaattccggggatccgtcgacc
P8	tgttctggattggcgcgaagatgcgtaaccgtaagcaggaagttgctgagagcggtggcggtggc
P9	gtcactttccgccatttgctg

P10	ggatgccagcctgagagc
P11	aagccgatgccagatttacg
P12	ggtatagatttcaacattggcc
P13	gtgcctttaacgctgctgtg
P14	tacttgtgctggacgtagcg
P15	caagccgaagcaatggtgac
P16	cagcggtctggacagtgaaac

Supplemental material references

1. Govers SK, Campos M, Tyagi B, Laloux G, Jacobs-Wagner C. 2024. Apparent simplicity and emergent robustness in the control of the Escherichia coli cell cycle. Cell Systems 15:19-36.e5.

2. Zimmermann L, Stephens A, Nam S-Z, Rau D, Kübler J, Lozajic M, Gabler F, Söding J, Lupas AN, Alva V. 2018. A Completely Reimplemented MPI Bioinformatics Toolkit with a New HHpred Server at its Core. J Mol Biol 430:2237–2243.

3. Bittrich S, Segura J, Duarte JM, Burley SK, Rose Y. 2024. RCSB protein Data Bank: exploring protein 3D similarities via comprehensive structural alignments. Bioinformatics 40:btae370.

4. Ke N, Landgraf D, Paulsson J, Berkmen M. 2016. Visualization of Periplasmic and Cytoplasmic Proteins with a Self-Labeling Protein Tag. J Bacteriol 198:1035–1043.

5. Cherepanov PP, Wackernagel W. 1995. Gene disruption in Escherichia coli: TcR and KmR cassettes with the option of Flp-catalyzed excision of the antibiotic-resistance determinant. Gene 158:9–14.
